# Muscle Involvement in Amyotrophic Lateral Sclerosis: Understanding the Pathogenesis and Advancing Therapeutics

**DOI:** 10.3390/biom13111582

**Published:** 2023-10-26

**Authors:** Elisa Duranti, Chiara Villa

**Affiliations:** School of Medicine and Surgery, University of Milano-Bicocca, 20900 Monza, Italy; e.duranti@campus.unimib.it

**Keywords:** skeletal muscle, amyotrophic lateral sclerosis, muscle atrophy

## Abstract

Amyotrophic lateral sclerosis (ALS) is a fatal condition characterized by the selective loss of motor neurons in the motor cortex, brainstem, and spinal cord. Muscle involvement, muscle atrophy, and subsequent paralysis are among the main features of this disease, which is defined as a neuromuscular disorder. ALS is a persistently progressive disease, and as motor neurons continue to degenerate, individuals with ALS experience a gradual decline in their ability to perform daily activities. Ultimately, muscle function loss may result in paralysis, presenting significant challenges in mobility, communication, and self-care. While the majority of ALS research has traditionally focused on pathogenic pathways in the central nervous system, there has been a great interest in muscle research. These studies were carried out on patients and animal models in order to better understand the molecular mechanisms involved and to develop therapies aimed at improving muscle function. This review summarizes the features of ALS and discusses the role of muscle, as well as examines recent studies in the development of treatments.

## 1. Introduction

Amyotrophic lateral sclerosis (ALS), which was first described by Charcot in 1874, is an extremely debilitating condition characterized by the progressive loss of upper motor neurons (MNs) in the cortex and lower MNs in the brainstem and spinal cord. Individuals with ALS experience a range of clinical symptoms, including muscle wasting, difficulties with speech and swallowing, fasciculations, altered reflexes, and spasticity. Unfortunately, patients face a grim prognosis, with death occurring within 2–5 years of diagnosis, primarily as a result of respiratory complications [1,2]. ALS is classified as a proteinopathy because protein aggregates are found in the affected MNs. The majority of ALS cases are classified as sporadic (sALS), while approximately 5–10% of cases have a familial (fALS) pattern. Both forms of the disease share similar pathological features, such as the presence of protein inclusions and neurological symptoms although there are differences between patients carrying different mutations. To date, more than 50 genes have been implicated in the etiology of ALS [3], but fALS is primarily associated with mutations in the *SOD1*, *TARDBP*, *FUS/TLS* and *C9ORF72* genes, encoding for the Cu/Zn superoxide dismutase-1, transactive response DNA-binding protein 43 (TDP-43), fused in sarcoma/translocated in liposarcoma and intronic GC repeat expansion in the chromosome 9 open reading frame 72, respectively [1]. These genes are involved in a wide range of cellular pathways, including mitochondrial dysfunction, excitotoxicity, autophagy with loss of protein homeostasis, inflammation, DNA damage repair, aberrant RNA metabolism and impaired intracellular trafficking [4].

Different criteria can be used to classify ALS. Firstly, environmental and lifestyle factors have been researched, revealing a greater prevalence of ALS in certain groups such as athletes, implying a possible association between physical activity and the disease. This link, however, has yet to be fully confirmed. Secondly, ALS can be distinguished based on the involvement of the first or second MN, resulting in different clinical onsets, either bulbar or spinal. Finally, genetic causes play a significant role in ALS, with certain gene mutations contributing to the disease development [1,2].

Muscle tissue possesses remarkable regenerative abilities, allowing it to repair severe injuries successfully [5]. In the context of ALS, a neuromuscular disease characterized by pathological alterations in muscle, this regenerative mechanism appears to be impaired. Manzano et al. previously demonstrated a decrease in the differentiation capacity of myoblasts into myotubes and fibers, as well as myogenic defects in mouse myoblasts carrying *SOD1* or vesicle-associated membrane protein-associated protein B (*VAPB*) mutations [6,7,8]. Specifically, VAPB mutants have low ATP levels and promote fat infiltration into myofibrils, which may suggest that metabolic changes are activated to compensate for mitochondrial dysfunction [9]. Moreover, numerous cases of ALS patients have shown signs of defective muscle regeneration, which is characterized by aberrant fusing of immature muscle cells and the formation of atypical myogenic patterns [10]. Muscle atrophy in ALS has traditionally been considered to be a result of denervation, in which the loss of nerve signals causes muscle deterioration. Recent research, however, has uncovered a more complex scenario. Muscle progenitor cells were activated in ALS patients’ biopsies even before the onset of clear neurological symptoms, indicating that muscle involvement in ALS is not only a result of denervation but also involves complex interactions within the muscle tissue itself [6,11]. 

No treatments exist to fully repair muscle damage in ALS and the available therapeutic options are mostly palliative, aiming at delaying disease progression. Two Food and Drug Administration (FDA)-approved medications are currently prescribed to patients, such as riluzole and edaravone, with the latter only being approved in some countries [12,13]. Additional therapies, including a combination of sodium phenylbutyrate and taurursodiol (AMX0035, also known as Relyvrio or PB/TURSO) and tofersen, have recently approved by the FDA and have shown promise in helping delay ALS patients’ functional decline [14]. In this comprehensive review, we delve into the complicated involvement of muscle tissue in ALS, examining the more recent studies to investigate potential therapeutic options that could restore muscle function and improve the quality of life for individuals living with ALS.

## 2. A Brief Overview of ALS Genetics

In addition to a marked phenotypic variability, ALS is also characterized by genetic heterogeneity. Therefore, studying the genetic mutations associated with the disease will link the molecular and cellular mechanisms of the disease, thus improving our understanding of ALS pathogenesis. Approximately thirty years ago, the first gene discovered to be associated with ALS, especially with the familial forms, was *SOD1* [15], accounting for 20% of fALS cases. The most common mutations include G93A, A4V, H46R, and D90A, which are usually inherited in a dominant trait, except for the last one, which also shows a recessive inheritance pattern of transmission, but only in the Scandinavian population [16,17]. The discovery of this gene led to the identification of different pathways involved in ALS progression through the generation of SOD1-related models. SOD1 protein aggregations produce a toxic gain of function resulting in neuronal loss, a primary cause of the disease [18,19]. An insoluble SOD1 ubiquitin-positive inclusion body within the MN is the signature pathological feature [20]. The mutant SOD1 protein forms cytoplasmic aggregates that can cause neuronal cell death by trapping essential cytoplasmic proteins required for neuronal survival, impeding the ubiquitin–proteasome system (UPS), resulting in chaperone protein depletion, damaging mitochondria, and impeding cytoskeletal or axonal transport [21].

In 2006, the genetic landscape of ALS was further enriched with the identification TDP-43 protein as a critical pathological component in cellular inclusions associated with both sALS and non-SOD1 fALS [22]. This protein is involved in gene transcription and RNA processing, and it is recognized as a primary cause of ALS [23], providing evidence that mutant TDP-43 can trigger neuronal degeneration and lead to ALS. Most of the *TARDBP* mutations are primarily concentrated in the glycine-rich C-terminal region and play a critical role in the movement between the nucleus and cytoplasm, tendency to form aggregates, and interactions between proteins [24]. Besides ubiquitination, the excessive phosphorylation of TDP-43 plays a significant role in the formation of protein aggregates in the pathogenesis of ALS. Specifically, in specimens taken from ALS patients, there is evidence of pronounced phosphorylation occurring at the C-terminal region of TDP-43, thereby promoting its aggregation [25].

Later advances in ALS genetic research led to the discovery of mutations in another RNA/DNA-binding protein, namely FUS/TLS as a primary cause of fALS [26]. FUS regulates mRNA transport towards dendrites and supports synaptic plasticity upon activation of glutamate receptors [27]. A common genetic characteristic with TDP-43 is that pathological mutations are located in the C-terminal part of the NLS domain [28], and all of this disrupts the cellular localization balance of these two proteins [27,29]. The genetic heterogeneity typical of ALS also reflects clinical variability. For instance, in both TDP-43-linked ALS and FUS-linked ALS, the age and the disease onset as well as clinical phenotype, exhibit variability, similar to sporadic ALS. Incomplete penetrance has been documented for several of these mutations [23,27]. Most patients with mutations in *TARDBP* and *FUS* genes develop the classic phenotype of ALS, characterized by typical pathological features of the disease.

In 2011, the pathogenic expansion of GGGGCC(G4C2) hexanucleotide repeats in the first intron of the *C9ORF72* gene was discovered to be the leading genetic cause of both ALS and frontotemporal dementia (FTD) [30]. Three pathogenic mechanisms have been suggested: (1) loss of C9ORF72 function due to decreased *C9ORF72* mRNA and protein expression; (2) toxicity from the bidirectionally transcribed repeat-containing RNAs, such as those mediated by the sequestration of key RNA-binding proteins (RBPs) into RNA foci, and (3) toxicity from production of repeating dipeptides through unconventional translation of these transcripts [31]. Alterations in several downstream cellular functions, including nucleocytoplasmic transport and autophagy, have been implicated. Significant UPS impairment is present in patients carrying *C9ORF72* mutations and ubiquitin-positive neuronal inclusions are their characteristic pathological features [32].

## 3. Main Pathways Altered in ALS Muscle

To date, significant progress has been made in ALS research through the establishment of mouse models resembling genetic features related with the disease [33]. These models are useful in unraveling the mechanisms behind ALS, particularly by examining different mutations in ALS-related genes. Among them, SOD1-G93A is the commonly used transgenic model that mimics the early onset and rapid degeneration seen in people with the G93A mutation, resulting in MN loss and muscle atrophy [34]. To gain a better understanding of ALS pathogenesis, researchers have focused on knocking down several genes associated with the disease to decipher their functions [35]. For example, knocking down *Sod1* in skeletal muscle did not result in the typical muscle atrophy observed in ALS, but it did lead to increased weakness and the presence of amorphous regenerating fibers [36]. Furthermore, reduced muscle mass was observed in an animal model with TDP-43 overexpression in muscle, due to increased cellular stress, specifically the unfolded protein response system (UPR) [37]. These experiments highlight the importance of gene function in targeted tissues, as different mutations can result in distinct events in the various tissues affected by the disease. As mentioned earlier, ALS patients exhibit an altered muscle phenotype characterized by progressive atrophy and wasting, placing ALS in the same category as other neuromuscular diseases. However, to distinguish ALS from typical muscular pathologies, it is crucial to remember that such alterations occur not only in the muscle but also in the CNS, particularly in MNs [38,39,40]. Some of these models manifest a phenotype resembling ALS in both muscle and CNS, with overexpression of SOD1 and related mutations inducing muscle atrophy and MN degeneration. Notably, in the SOD1-G93A model, overexpressing insulin-like growth factor-1 (Igf-1) in muscle slows down disease progression by preventing neurodegeneration and muscle weakness [41]. In particular, IGF-1 plays a pivotal role as a growth-promoting factor, governing both constructive and degradative processes within the skeletal muscle. IGF-1 increases skeletal muscle protein synthesis via PI3K/AKT/mTOR and PI3K/AKT/GSK3β pathways that will be further discussed later [42]. These studies suggest that muscle may serve as a primary target in ALS, indicating that factors derived from muscle fibers are crucial for neuron survival. 

Direct research on ALS patients demonstrates an early involvement of muscle in the disease development. Muscle atrophy resulting from denervation is not the only observation; pathological features caused by fiber inflammation are also present, implying that muscle atrophy is more than just a result and that myogenic defects play a vital role in disease onset [43,44]. 

The UPS E3 muscle-specific ubiquitin ligases ring finger 1 (MURF1) and F-box muscle atrophy (atrogin-1/MAFBX) are now being studied. These markers are consistently found in clinical cases of muscular atrophy, and their expression is increased in patients experiencing atrophy [45,46]. Another study emphasizes the importance of AKT protein function, as low expression correlates with a worse prognosis and survival in ALS patients. Muscle biopsies of ALS patients show lower levels of many proteins, including IGF-1, while the β IGF-1 receptor subunit (IGF-1Rβ) exhibits a significant increase, leading to the dysregulation of AKT expression in ALS muscle [33,47]. This is significant because the IGF-1/PI3K/AKT signaling pathway promotes muscle hypertrophy, and its inhibition leads to muscle atrophy, exacerbating the condition of affected tissue [48,49,50,51]. Therefore, the alterations in this signaling pathway and the disruption of AKT found in both patients and animal models have also revealed changes in the mammalian target of rapamycin (mTOR), which is regulated by AKT. Glass and colleagues clearly demonstrated that mTOR is a downstream target of AKT, and they showed in their study that it is essential for promoting muscle hypertrophy [52]. In other studies, an improvement in muscular atrophy in animal models with muscle damage has been observed after treatments with mTOR and the restoration of AKT levels [53]. However, these cellular signaling pathways in skeletal muscle have been comprehensively examined in a recent review by Yoshida and Delafontaine [42].

As mentioned earlier, it has been found that these alterations to the signaling pathways described above also have an impact on protein management processes, specifically proteostasis. This mechanism is also impaired in ALS, resulting in the loss of key proteins required for muscle myogenesis and regeneration, ultimately muscle mass loss. We have previously explained that UPS alterations involved in the AKT pathway result in favoring muscle atrophy and morphological and functional changes. Another protein degradation process within proteostasis is autophagy. Unfortunately, it has received less attention than the UPS in ALS-affected muscles. In fact, few research studies have focused on the autophagy process, which involves the delivery of substrates delivered to lysosomes and their degradation by proteolytic enzymes [54]. Autophagy in muscles is a finely regulated process that is affected by physiological conditions and any metabolic stress the cell may undergo. As mentioned, in ALS, this process is disrupted, and its altered activity is characterized by the inhibition of lysosome–autophagosome fusion, which can lead to pathological states and mutations in autophagic genes. All of this has been proposed as one of the primary causes of typical ALS protein aggregation development in skeletal muscle [55]. At the molecular level, muscle fiber atrophy can be attributed to the presence of protein aggregates often associated with the disease, including TDP-43, FUS, or SOD1, which can be detected not only in the cytoplasm of neurons but also within muscle cells [1,30,56,57,58,59]. 

However, more detailed insights into this aspect will be provided in the next paragraphs.

## 4. Mitochondrial Dysfunction in ALS Muscle

**Figure 1:**
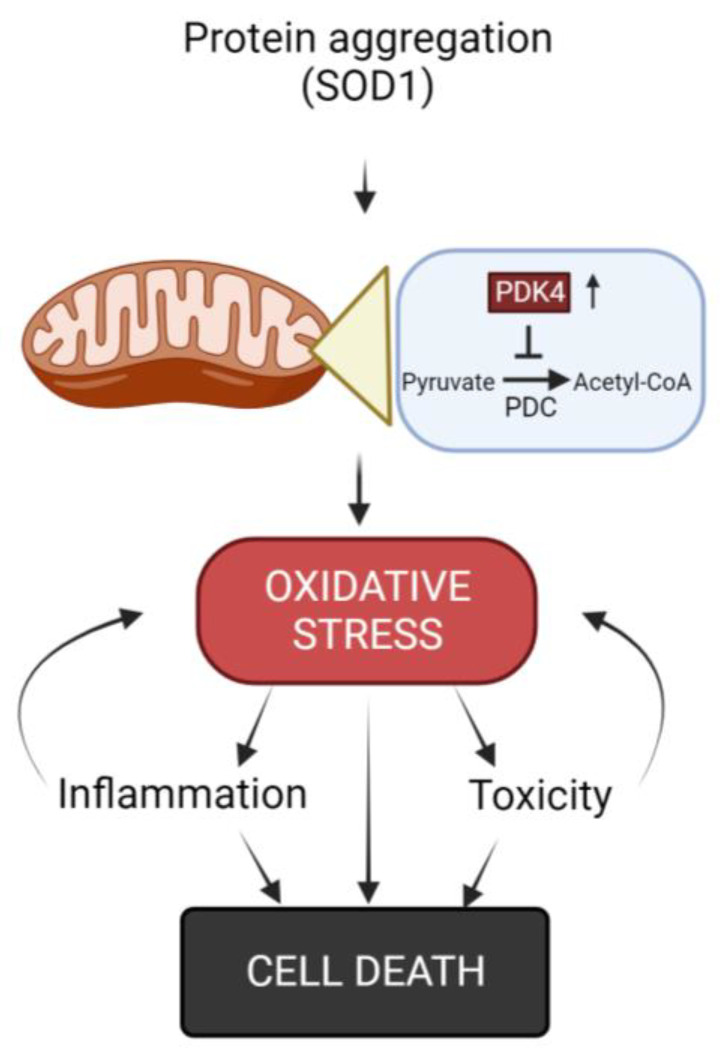
Diagram of oxidative stress and its involvement in the disease development.

As previously stated, oxidative stress is a process involved in muscle damage seen in ALS patients (Figure 1) [60]. Manganese SOD (Mn-SOD) is a mitochondrial enzyme that detoxifies anion radical superoxide, molecular oxygen and hydrogen peroxide [61]. This is a cellular mechanism aimed at counteracting ROS and the cellular damage it produces. Vielhaber and colleagues demonstrated that Mn-SOD deficiency in human muscle tissue appears to be a process involved in sporadic ALS [62,63]. All of this was later confirmed by subsequent investigations, including one by Xiao et al. which demonstrated an interesting study where it is shown how reactive oxygen species (ROS) accumulation and oxidative stress were found in the skeletal muscle of both ALS patients and also SOD1-G93A transgenic mice [64,65]. These animals, in particular, had cyclophilin D (CypD) overexpression, which resulted in ROS production [65,66].

Excessive ROS production has been reported to cause mitochondrial DNA (mtDNA) damage, resulting in respiratory chain problems in the mitochondria of skeletal muscle cells from fALS patients [62,67], demonstrating thus a deficient mitochondrial respiratory chain and ATP generation. Furthermore, various studies have hypothesized and brought to light that the protein aggregation, which is common in ALS and observed in muscle, can aggravate oxidative stress, since the control of the correct protein conformation requires a lot of energy, consequently forcing the mitochondrial oxidative phosphorylation and the generation of ROS [4,68]. 

The increase in pyruvate dehydrogenase kinase 4 (PDK4) levels, together with the inhibition of the pyruvate dehydrogenase complex (PDH), which is crucial for the conversion of pyruvate into acetyl-CoA inside the mitochondria (a critical step in the energy production process within cells) and phosphofructokinase 1 (PFK1) is another mechanism that causes mitochondrial damage and alters muscle homeostasis. This process leads to the accumulation of glycogen and, as a result, an increase in ROS which aggravates the mitochondrial dysfunction [69,70]. In the context of ALS, some research has highlighted an increase in the expression of PDK4 in affected muscles. However, there are data from research on the effects of the administration of dichloroacetate (an inhibitor of PDK4 activity), to the mouse model SOD1-G86R which appears to promote glycolysis, protect muscle mitochondria and prevent muscle atrophy [69,70,71]. Ongoing further studies are aiming to better understand the role of PDK4 and its implications for muscle metabolism and the progression of ALS. Understanding these mechanisms could potentially lead to the development of targeted therapies to improve muscle function and slow down the progression of the disease in ALS patients.

## 5. Protein Accumulation Involvement in ALS Muscle

**Figure 2:**
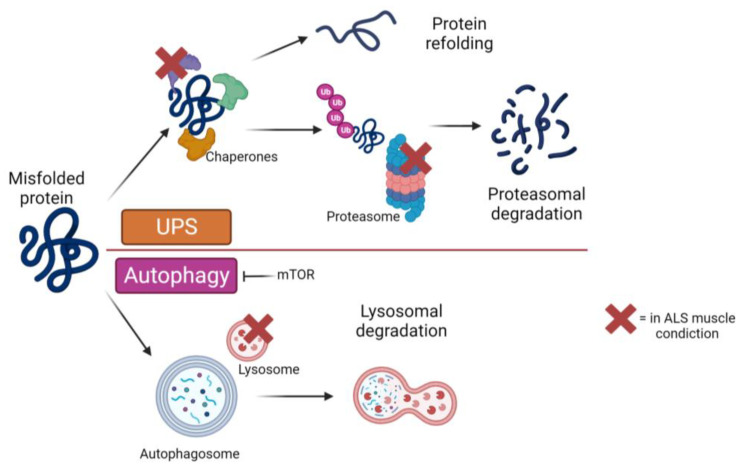
Schematic representation of proteostasis pathways that have failed in muscle with the ALS phenotype (marked with a red cross).

In the context of ALS, it is critical to explore the numerous processes involved in muscle degeneration, as they play a crucial role in maintaining cellular homeostasis. These mechanisms have been extensively studied in relation to the pathology of MNs. As shown in Figure 2, one such process is the accumulation of proteins in the cytoplasm and the disruption of protein degradation (Figure 2). The UPS is responsible for degrading soluble proteins that are misfolded [72]. Studies in animal models [73,74] and humans [75] indicated that proteasome function is strongly impaired in ALS tissues. Autophagy, an intracellular mechanism responsible for the degradation of misfolded proteins, is particularly relevant in this context, even though, as already mentioned in the previous paragraph, more research is still needed in muscle tissue.

A notable study conducted by Oliván and colleagues shed light on the alterations in autophagic factors observed in mice overexpressing mutant SOD1. The researchers found increased expression of LC3, p62, and Beclin-1 (BECN1) in the muscle of these mice [76]. Other comprehensive studies have corroborated these findings [77,78,79]. Furthermore, in wild-type mice, autophagy is appropriately activated in response to cellular stress, resulting in the formation of autophagosomes and other components involved in protein degradation. However, in SOD1 mutant mice, the normal autophagy process is disrupted, possibly due to the dissociation of the BECN1 autophagy promoter and caspase 3 [77].

Another key player in the autophagic process is mTOR, a kinase that regulates various cellular processes, including protein synthesis. Studies have demonstrated that mice with ALS mutations exhibit reduced mTOR activity, which is associated with impaired autophagy. Further research into this pathway and its connection with mTOR has demonstrated that additional inhibition of mTOR with rapamycin exacerbates muscular atrophy and weakness [80,81]. 

Overexpression of the KH-domain-containing ankyrin-repeat protein enhanced autophagosome degradation in a fly model bearing the mutant FUS frequently linked with familial ALS [1]. This enhancement in autophagy contributed to an increased protein aggregation state in muscle, ultimately leading to an improvement in muscle condition compared to the untreated model [82].

TDP-43 is another aggregation protein closely associated with ALS, specifically the TDP-25 fragment. Pathological accumulation of these aggregates has been observed in the cytoplasm of muscle cells resembling the ALS phenotype. Although these aggregates are smaller than those found in MNs, they still have the ability to disrupt autophagy processes [83]. Moreover, studies analyzing muscle biopsies of ALS patients have consistently found abundant inclusions of the total phosphorylated TDP-43 form [84,85]. Finally, the ALS research has revealed the involvement of various processes in muscle degeneration, highlighting their crucial role in cellular homeostasis. Autophagy, protein degradation, and the dysregulation of key factors such as LC3, p62, Beclin-1, mTOR, SOD1 and TDP-43 have all been identified as significant contributors to disease progression. Understanding these mechanisms provides valuable insights into the pathogenesis of ALS and offers potential targets for therapeutic interventions. More research in this field is needed to unravel the complex interactions and develop novel strategies to combat ALS and mitigate the debilitating effects of muscle degeneration.

## 6. The Neuromuscular Junction in ALS: Understanding Pathogenesis and Therapeutic Potential

The neuromuscular junction (NMJ) is a complex connection that bridges the ends of MNs with muscle fibers, allowing muscle contraction. Its intricate structure consists of more than just MNs and muscle fibers [86]. It also involves Terminal Schwann cells (TSCs) and kranocytes, which contribute to the general stability and maintenance of the NMJ [87,88]. TSCs, for example, are responsible for regulating NMJ stability and facilitating repair processes, while kranocytes respond to any insults the NMJ. It is essential for all these components to function harmoniously to maintain the integrity of the NMJ [88,89].

The dismantling of the NMJ assumes a significant role in the development of the pathological phenotype, primarily through a process known as axonopathy [90]. MN degeneration triggers a cascade of pathogenic processes, including denervation of NMJ, which creates an environment devoid of regeneration capacities and, as a result, hampers successful muscle reinnervation [91]. Interestingly, it has been reported that repression of mutant TDP-43 expression leads to axon regeneration by the remaining MNs [92]. Various alterations in synaptic properties and different components of the NMJ have been reported in ALS, contributing to the observed dysfunction and pathology. Remarkably, this denervation process unfolds as a complex and dynamic phenomenon, occurring even before the onset of noticeable symptoms. 

Research has shed light on the influence of alterations in pre-synaptic components, particularly TSCs, on NMJ stability and regenerative potential. Notably, TSCs in ALS cases exhibit abnormal morphology and impaired sprouting abilities [88,89,90,93]. Delving into the role of the NMJ in the pre-synaptic component, Lepore and colleagues reported that there are various events contributing to creating an environment in which regeneration is compromised, ultimately leading to NMJ denervation, a characteristic feature of ALS as previously mentioned [39]. Mice carrying the *SOD1* G37R mutation showed that motor units are disassembled incorrectly in this mutant model. This study demonstrated that the process begins gradually and specifically, but later triggers axonal degeneration [91]. From this evidence, it can be speculated that the severe denervation typical of ALS is due to this entire system’s inability to consistently perform the proper reinnervation process. Also, the location of the NMJ can influence the process: it has been found that those located on axons in distal branches are more likely to encounter issues compared to those in proximal areas [91]. Another important piece of data supporting the importance of proper reinnervation comes from studies that show that after denervation due to nerve incision, the repair process at the terminal end of the cell is compromised in *SOD1* G93A mice before the onset of the disease [90,94]. Emerging evidence suggests that terminal TSCs do not efficiently contribute to synaptic repair in SOD1-G37R animal model SOD1 or ALS patients [93]. In this case, they were analyzed after experimental partial denervation, and it was revealed that TSCs are unable to promote reinnervation [90]. Finally, the lack of effective synaptic repair generates an inappropriate environment for terminal nerve growth, which contributes to NMJ degeneration and the progression of ALS.

The post-synaptic aspect involves modifications in the signaling pathways that affect the stability of nicotinic acetylcholine receptors (nAChR) clusters, hence influencing the overall stability of NMJ [95]. Dysregulation of specific proteins, such as FUS, often detected in ALS, can contribute to the destabilization of the NMJ endplates [95]. Additionally, secreted factors and signals originating from satellite cells (SCs) are critical in guiding axonal growth and promoting NMJ regeneration. Importantly, NMJ undergoes dramatic functional, morphological, and molecular alterations during aging, leading them to ultimately degenerate [96].

Looking more closely at this section, denervation causes small spots of fragmentation in the synaptic cleft, thereby worsening system function. All of this has been demonstrated in both murine models and in humans [97,98,99]. There are distinct molecular mechanisms underlying the regulation of the nAchR system at the subsynaptic level in innervated muscle fibers. Similarly, the proteoglycan Agrin, produced by nerve cells and TSCs, has emerged as a regulator of correct AchR function through the LRP4-MuSK receptors [88,100,101]. Furthermore, because of its propensity to interact with transcription factors, FUS has been found to be a component of the nAchR regulatory pathway. This latter finding is significant in understanding why patients and murine models with the FUS mutation exhibit synaptic plaque instability [95].

Moreover, SCs play an important role in NMJ homeostasis because they produce factor and activate cellular signals that can guide axonal activity and facilitate reinnervation following insults [102]. A branch of study that has demonstrated Neuropilin1/Sema3 and HGF/Sema3A to be relevant in animal models with the SOD1 G93A mutation for the correct regeneration of nerve cells is indisputable evidence underlying muscle’s role in the disease. Muscle tissue, being the primary source of SEMA3A, is crucial for MN regeneration, since it cannot occur without its secretion [103]. All of this has been proven by studies that found a significant presence of SCs in models with peripheral nerve injury to repair the NMJ during the regeneration phase [88]. In parallel, it has also been observed in models lacking SCs, where NMJ regeneration was compromised and degeneration was favored [96,104].

Given the importance of NMJ components and their communication in ALS pathogenesis, strategies aimed at preserving NMJ function and structure may hold promise for ALS treatment. However, it is important to note that ongoing studies are actively investigating these avenues in order to deepen our understanding of the underlying mechanisms and develop effective therapeutic strategies. 

In summary, ALS exhibits a phenotype with the degeneration of MNs and muscles arising at the NMJ [105,106]. Dysfunctions of both pre- and post-synaptic NMJ components contribute to NMJ instability and the disease progression. As a result, attempts to preserve NMJ function and structure have the potential to mitigate the burden of ALS.

## 7. Muscle Regeneration in ALS: Insights from Satellite Cells and Myogenic Factors

In the previous chapters, we explored different pathways that could cause muscle injury, ultimately leading to the development of muscles with amorphous morphology in ALS patients due to erroneous muscle regeneration. Here, we go deeper into the complex process of muscle regeneration.

**Figure 3:**
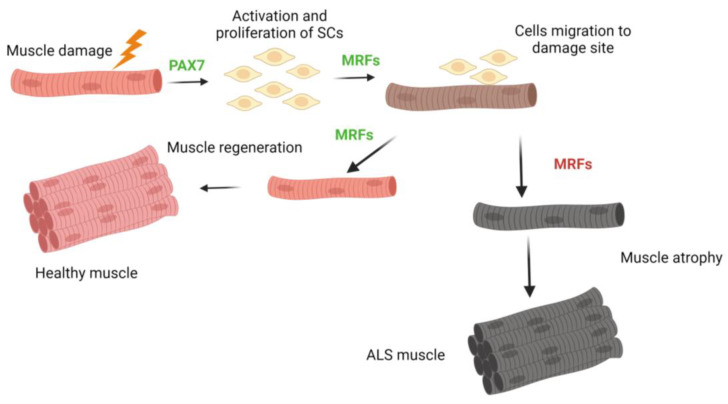
The muscle regeneration process occurs after an injury. When the expression of MRFs is altered, muscle damage is not properly repaired, leading to the typical onset of muscle atrophy found in ALS.

The remarkable plasticity of skeletal muscle is a distinguishing feature, allowing it to adapt and modify its properties in response to the changing conditions [107]. Moreover, one of its key characteristics is the ability to repair and regenerate. As previously stated, muscle regeneration primarily depends on the activation of SCs, which are highly resilient cells located beneath the basal membrane of differentiated muscle fibers and endowed with exceptional endurance [108] (Figure 3). In adults, these cells exist in a quiescent state, expressing the paired-box transcription factor Pax7, which is essential for the survival, maintenance, and myogenic potential of SCs [109,110]. Pax7-positive SCs are activated when muscle tissue is subjected to exercise-induced lesions or muscular pathologies, as shown in Figure 3.

SCs begin the process by proliferating and co-expressing the myogenic differentiation protein 1 (Myod1). These activated SCs then upregulate the expression of myogenin (Myog) and merge together to form myotubes, thereby regenerating the damaged muscle fiber within the muscle tissue [110,111,112]. The myogenic regulatory factors (MRFs) are capable of forming both homodimers and heterodimers. Through their basic helix–loop–helix (bHLH) domain, they bind to specific DNA sequences known as E-boxes (CANNTG), which are ubiquitously present in the promoter and enhancer regions of various genes, whether muscle-specific or non-muscle-specific [110]. In SOD1-G93A mice, mRNA levels of *Pax7*, *Myf5*, *MyoD1*, and *Myog* were all elevated during the advanced stages of the disease. This upregulation represents the muscle’s attempt to renew itself by increasing MRFs transcription. However, when the protein level is examined, only a minimal response is observed [113]. It is suggested that the accumulation of increased MRF transcripts may be attributed to post-transcriptional mRNA stabilization. New in-depth investigations are required to truly and clearly understand why there is an increase in mRNA expression and a decrease in key proteins essential for appropriate myogenesis in muscle. It can be hypothesized that the cause for some of these proteins is due to the pathogenic nature of the disease, notably malfunctioning and alterations in protein degradation mechanisms and their half-life. These changes may result in aberrant cellular communications, which increase mRNA production in an attempt to compensate for or regulate the abnormalities at pathological protein levels. Unfortunately, this does not solve the problem and instead creates pathological imbalances. However, this is merely a theory that needs to be explored and investigated to determine whether it is correct or what genuinely causes this imbalance in ALS.

Furthermore, various studies have demonstrated that the susceptibility of different types of muscle fibers to SOD1-G93A toxicity can vary. Notably, a distinction between fast- and slow-twitch-fiber responses has been observed in SOD1-G93A mice [39,114,115,116]. This discrepancy in sensitivity could be attributable to cellular consequences resulting from the mutant SOD1 toxicity. 

In the muscle biopsies from ALS patients, it has long been noted that there is an increase in euchromatin inside SCs, although their fraction remains unchanged [33,117]. Recent studies corroborate this observation, demonstrating a decrease in the expression of myogenic markers Pax7 and Myf5, as well as Pax7-positive SCs that do not express MyoD. These findings suggest an in vivo activation of SCs in ALS patients’ cells, but without progression through the myogenic program [118]. It has also been confirmed that SCs derived from ALS are unable to properly differentiate when cultured in vitro. This is demonstrated by their abnormal myotube morphology and lower expression of the MYF-4 isoform 1 heavy chain gene and protein compared to control samples [6,11]. As previously mentioned, some noteworthy members within the category of key MRFs myogenic are the MyoD and Myf5 transcription factor proteins, which are critical in beginning myoblastic commitment. In addition, MRF4 (also known as Myf6) and myogenin are required for maintaining myotube differentiation [112]. Pax7 directly activates MyoD and Myf5 transcription. Myoblasts, which are derived from myotome cells produce bHLH proteins and can proliferate when stimulated by growth stimuli. The absence of these components causes a halt in cell proliferation, fibronectin release, and integrin receptor activation. The fibronectin-integrin adhesion signal is required for myoblast differentiation to begin. Cell-to-cell recognition causes the cell cycle to be disrupted [110]. Myogenic stem cells expressing Pax7, referred to as SCs, are located in the region between the basal lamina and the sarcolemma of connected myofibers, playing a critical role in promoting adult muscle growth. These cells have the ability of self-renewal and, when activated, can move from a latent state to generate actively dividing myoblasts by restarting the cell cycle [119]. Myotubes isolated from patients with ALS had lower levels of fast, slow, and neonatal myosin heavy chain (MHC) proteins than myotubes derived from healthy people. Furthermore, there is a significant decrease in the levels of the Myog protein in ALS myotubes [6,11]. Muscle biopsies from ALS patients and healthy control participants reveal a similar absolute amount of satellite cells when stained with Pax7. There are extremely few nuclei positive for Pax7 but negative for Myog in ALS investigations [11]. These findings in muscle cells suggest that SCs have entered a senescent state and that there is a problem with the signals essential for correct and effective myogenesis. As a result, the muscle cannot repair and renew as well as it can under normal physiological conditions, resulting in severe muscular atrophy. Nonetheless, further research into muscle and its biology is required to definitively resolve this issue.

## 8. RNA Processing and Involvement in ALS Pathology

Many of the ALS-associated proteins, such as TDP-43 and FUS, are RBPs that contain one or more RNA recognition motifs critical for their normal biological function [1]. These proteins often possess low-complexity or prion-like domains that enable phase separation into microdomains where RNA processing occurs. In fact, many ALS-associated proteins accumulate in RNA foci, such as cytoplasmic stress granules or nuclear paraspeckles, which are membrane-less organelles strongly implicated in ALS [32,120]. This noteworthy characteristic can potentially explain the broad-ranging impact on RNA metabolism resulting from ALS-causing mutations. However, it is important to note that not all ALS genes inherently encode RBPs. Certain mutations in *SOD1* and *C9ORF72* can efficiently sequester RBPs or other gene products, thereby significantly impairing their normal biological function and causing severe dysregulation of RNA metabolism [121]. While our understanding of RNA metabolism in ALS has undeniably improved in recent years, our current knowledge regarding the mechanistic involvement of these processes in the observed skeletal muscle pathology in ALS, or more broadly in the etiopathogenesis of the disease itself, remains relatively limited.

With regard to RBPs, research has consistently highlighted that mislocalization and accumulation of TDP-43 can severely disrupt RNA processing in neurons. TDP-43 primarily localizes in the nucleus of healthy cells, where it regulates transcription and pre-mRNA splicing [1]. However, in ALS, it undergoes delocalization to the cytoplasm, resulting in dysregulation of its normal nuclear targets [122]. In muscle tissue, TDP-43 plays a critical role in in vitro differentiation and is necessary for in vivo muscle regeneration [123]. Notably, the Drosophila ortholog of TDP-43 actively promotes the development of neuromuscular connections, and its decrease correlates with a decline in motor capacity, supporting its active role in proper muscle function [124,125]. Recently, compelling evidence has emerged revealing that TDP-43 produces aggregates containing mRNA during normal skeletal muscle regeneration [123]. This mRNA specifically encodes sarcomeric proteins, but interestingly, it becomes depleted in fully mature myotubes. Collectively, these findings strongly suggest that conditions associated with the TDP-43 mislocalization, a hallmark characteristic of ALS, possess the potential to disrupt the delicate balance of assembly and clearance of these structures within skeletal muscle.

Similarly to TDP-43, FUS protein plays a pivotal role in mRNA splicing and localization, as well as in stress granule formation and other aspects of RNA metabolism. However, unlike TDP-43, FUS alterations do not exert any discernible effect on MN function [126], thereby implying that FUS-dependent motor degeneration in ALS potentially involves distinct underlying mechanisms [95,127]. Notably, the mutated form of FUS exerts toxic effects on muscle cells, which can be attributed in part to defective transcriptional regulation of AChR subunits. Moreover, FUS is an essential nuclear component that largely participates in the stress-induced silencing complex, which primarily facilitates the nuclear transport of small nucleotide sequences, such as micro(mi)RNAs [128]. Additionally, FUS plays an important role in miRNA-regulated gene silencing by successfully interacting with miRNA core-binding argonaute (AGO) proteins [129]. However, the precise extent to which these processes are adversely affected by FUS mutations in the context of ALS within skeletal muscles still remains to be fully elucidated. 

## 9. Role of Physical Activity as a Therapy for ALS: Potential Benefits

Muscle is a tissue that can easily respond to a variety of conditions, such as nutritional conditions, hormonal responses, and other factors that can influence its structure. Above all, muscles adapt to physical exercise by undergoing physiological changes that result in health advantages [130]. Regular physical activity gives numerous benefits to patients suffering from neuromuscular diseases, including ALS [131]. As a result, it is important to evaluate clinical cases and consider if exercise therapy could slow down muscle degeneration and preserve the NMJ integrity in patients. Researchers are evaluating this hypothesis because physical exercise may activate muscle metabolism pathways, increase the consumption of muscle glucose and promote proper muscle regeneration [132,133]. Exercise has antioxidant effects, as well as increasing mitochondrial biogenesis, and neurogenesis [134,135,136,137,138]. Tseng et al., for example, found higher levels of circulating antioxidants in MRL/MpJ mice, an excellent mouse model for studying molecular and cellular pathways during tissue regeneration because of its high capacity for tissue repair. Interestingly, they also discovered that deletion of the *Sod1* gene affected the myogenic capacity of these mice, suggesting a possible link between gene mutations in ALS and the regulation of muscle regeneration [139]. For this reason, research is ongoing to further investigate and develop therapeutic approaches based on this therapy. This is because physical activity could also be toxic if applied incorrectly as therapy, as high levels of the same stimuli have negative or even toxic effects [140]. Regular moderate-intensity training reduces cell damage caused by inflammatory processes produced by ROS [141,142,143] and protects against aging-related changes in muscle mitochondrial function [144,145,146]. Continuous high-intensity training, on the other hand, has been shown to have opposite effects [147,148].

In ALS animal models, only moderate-intensity physical activity improves the phenotype of SOD1-G93A mice [149,150,151,152,153]. There are also studies on the benefits of aquatic physical activities: swimming extends the lifespan of SOD1-G93A mice more than running [154,155]. This is thought to be because swimming includes the use of fast motor units, while running involves slow motor units [154,155,156,157,158,159]. A study on mice carrying the genetic mutation *SOD1* G93A examined the effects of physical exercise, particularly swimming, on the dysregulation of the BDNF/TrkB pathway [160,161]. Brain-derived neurotrophic factor (BDNF) is a protein that is secreted during muscle contraction and can act as both a neuroprotector as well as a neurotoxin. Hyperexcitability of neurons and consequent muscular contractions caused an over-secretion of BDNF in SOD1-G93A mice, which likely contributed to neurodegeneration by increasing glutamate toxicity [160]. 

Regarding humans, studies in ALS patients receiving traditional therapy and those undergoing exercise therapy have clearly demonstrated that physical activity can positively influence physical and muscle conditions [162]. Another study demonstrated that muscle contractions prevented muscle atrophy and increased muscle strength [163]. Therefore, physical activity has shown encouraging outcomes as a therapeutic option, particularly in terms of increasing patients’ quality of life. However, more in-depth research is required to develop precise and accurate therapy strategies that are actually beneficial.

## 10. Progress in ALS Research: Exploring Therapeutic Approaches and Promising Compounds

As previously stated, there are currently no effective therapies for the definitive treatment of ALS that can stop its clinical progression. Available treatments primarily focus on providing palliative care and include non-invasive ventilation (NIV), tracheotomy, and percutaneous endoscopic gastrostomy (PEG) [164,165]. This is due to the fact that ALS progresses in a variable manner until complete degeneration of MNs occurs [166]. 

### 10.1. Current Therapeutic Strategies

Despite numerous clinical trials over the last decade, only four drugs have been approved by the FDA with different regulatory guidelines around the world: riluzole, edaravone, the combination therapy AMX0035 and tofersen [14].

In Europe, the only drug approved in 1995 for ALS management is the glutamate release inhibitor riluzole. It can halt the disease progression and extend the median survival time of patients from three to six months but it shows adverse effects such as liver problems and diarrhea [13]. However, there is currently no evidence that riluzole improves MN function, lung function, fasciculations, or muscle function. Riluzole works by specifically inhibiting voltage-dependent sodium channels in the CNS, thus reducing calcium influx and glutamate-mediated cytotoxicity in the motor cortex and spinal cord [167,168]. It also appears to have antioxidant properties, as evidenced by its ability to reduce oxidative stress generated by various oxidizing agents [169]. 

In 2017, Radicava (edaravone) was approved by FDA for the treatment of ALS patients due to its potential therapeutic benefits highlighted by numerous studies [170,171]. It acts as a free radical scavenger, efficiently decreasing lipid peroxides in the same way that antioxidants like vitamin E and ascorbic acid do. It also targets hydroxyl radicals and peroxynitrites, as well as other ROS [172]. Edaravone protects neuronal, glial (microglia, astrocytes, and oligodendrocytes), and endothelial vascular cells from oxidative stress [173]. However, the exact mechanism of action by which edaravone exerts its therapeutic effects in ALS patients is still not fully understood.

In September 2022, the FDA also approved AMX0035 which is believed to decrease neuronal cell death by alleviating endoplasmic reticulum stress and mitochondrial dysfunction [174]. Patients receiving AMX0035 in the phase 2 CENTAUR clinical trial showed a significantly slower loss of physical function [175] and a longer median overall survival compared to placebo administration in a follow-up open-label extension [176]. Recently, in April 2023, tofersen was approved by FDA based on the results of the phase 3 VALOR clinical trial for the treatment of ALS patients carrying a mutation in the *SOD1* gene. Tofersen is an antisense oligonucleotide able to reduce the concentrations of SOD1 in cerebrospinal fluid (CSF) and the plasmatic levels of neurofilament light chain (NfL), a marker of neurodegeneration. Unfortunately, it did not improve clinical end points and it was associated with adverse effects. However, a phase 3 open-label extension study (ATLAS) is still ongoing to evaluate clinical benefits in patients with presymptomatic *SOD1*-ALS [177].

### 10.2. Current Clinical and Experimental Studies by Using New Possible Compounds

Researchers have explored various approaches to target muscle pathology as a means to counteract the degeneration of MNs in studies conducted and currently underway to identify compounds and potential medications that can alleviate and improve the condition of muscle tissue affected by ALS. One line of investigation focused on mitochondrial dysfunction, and early studies demonstrated that oral supplementation with creatine, which served as an energy source, had a beneficial effect on mutant SOD1 mice. When compared to control animals, these mice showed a significant improvement in their physical condition. Additionally, further studies exploring the administration of creatine found that it increased mitochondrial ATP production, hence enhancing muscle strength [178,179]. Despite these encouraging findings in muscle-related outcomes, creatine treatment has failed to produce optimal results in terms of improving overall longevity and slowing down the disease progression. As a result, researchers have continued to explore other compounds with possible therapeutic benefits [180,181]. In their studies, Groeneveld and Shefner also validated these results in ALS patients [182,183].

L-carnitine is one such compound that has shown promise in improving both the muscle pathology and the MN degeneration [184]. Furthermore, the importance of precise dietary programs cannot be overlooked. High-energy diets, especially those rich in lipids, have been shown to improve muscle strength, MN survival, and overall quality of life in ALS patients [185]. These studies reflect the continuous efforts to find compounds and therapeutic options that can successfully treat the muscular damage associated with ALS. While creatine supplementation has been found to improve muscular function [186], further research is needed to maximize its potential therapeutic advantages. Meanwhile, L-carnitine administration and adherence to specific energetic dietary regimens offers promising avenues for improving both muscle condition and overall disease progression in ALS patients [184].

Further research on mutant SOD1 mice has resulted in an increase in Igf-1 levels in the muscle. Normally, its levels are lower in ALS-affected muscles, contributing to muscle atrophy [187]. This increase has been found to improve muscle performance and increase MN survival [41]. Similar results were observed with the administration of dihydrotestosterone, which further elevated Igf-1 levels [188].

Current studies on the regulation of myogenic factors as a means of maintaining muscle health have revealed that gene transfer of myogenin into the muscle improves the condition of MNs. The same process using MyoD, however, worsened the condition [189]. Additional research has shown that reducing the response to stress and oxidative damage, as well as improving muscle contraction strength, can benefit ALS-affected muscles [190,191,192].

However, these compounds or studies on specific diets have not been proven to be effective in treating impaired autophagy in ALS. Therapeutic strategies modulating autophagy in ALS can be applied at different targets, mainly on improving its induction or the fusion of autophagosomes with lysosomes. As a result, extensive research has been carried out to identify chemical compounds capable of successfully promoting autophagy. Among them, rapamycin, a well-known pharmacological inducer of autophagy, has been the focus of several studies, although the impact of its administration on ALS varies significantly. In mice overexpressing TDP-43, it successfully improved MN function [193,194]. Conversely, in mice with *SOD1* mutations, it failed to yield any noticeable improvements in the best-case scenarios [195], and in the worst cases, it even resulted in toxic effects [196]. Therefore, future study must look deeper into the utilization of rapamycin while also exploring alternative molecules. In the past, it was assumed that the treatment outcomes could be influenced by the gender of patients, or the animal model used. As a result, researchers have investigated the role of hormones and how they affect ALS phenotype. Androgen injection has been shown to diminish autophagic activity, impair mitochondrial function, and increase oxidative stress. Treatment with female hormones, on the other hand, has had contradictory results [197]. Following research on tamoxifen and raloxifene, which were used to activate autophagy in ALS patients, it was found that these substances might provide protection in ALS-affected tissues [198,199]. Moreover, in female mice with mutant SOD1, reducing or eliminating estrogen production accelerate the course of ALS [200]. These findings also support evidence that premenopausal women are less susceptible to ALS than men. Additionally, they confirm the similar prevalence of ALS in both sexes during menopause [201,202,203].

The AChRs in muscle tissue which have been hypothesized to be potential therapeutic targets in this condition represent another critical topic in current research for muscle therapies. Studies have shown that the use of palmitoylethanolamide (PEA) minimizes desensitization of AChR currents after repeated stimulation, resulting in improvements in pulmonary function and quality of life [97,204,205]. PEA has also significantly increased the expression of the α1 subunit of AChRs and preserved NMJ functionality by reducing the decrease in ε-AChR currents. Additionally, the effect of riluzole on muscle AChRs has been observed, which appears to selectively block γ-AChRs [97,204,206]. Although these discoveries are intriguing and encouraging, they are still in their early stages, and further research is required to validate them (Table 1).

### 10.3. Stem Cell-Based Clinical Trials

Given their ability to self-renew and differentiate into any cell type, the use of stem cells has been proposed as an attractive alternative to conventional therapy with the aim of ameliorating muscle deterioration by replacing and repopulating lost or damaged MNs for transplant into ALS patients [207]. Different cell types, quantity of cells, and delivery routes/sites have been explored in ALS patients with only modestly encouraging results in terms of safety and efficacy. The majority of trials were small and single-center operations with diverse recruited patient populations, therapy regimens, and outcome measures, so they cannot be considered conclusive.

The first use of stem cells was applied in a pilot study with three ALS patients who received intrathecal injections of autologous peripheral blood stem cells (PBSCs). Among them, two individuals experienced improved speech and increased muscle strength for at least four months. Over the next twelve months, there were no side effects or acceleration of the disease, demonstrating the safety of this method [208]. PBSCs were also transplanted into the frontal motor cortex of enrolled patients in order to enhance the function of upper MNs. The median survival time was significantly longer compared with the control group and the scores on the Spitzer quality-of-life scale and the ALS Functional Rating Scale Revised (ALSFRS-R) were consistent over the course of the follow-up period, indicating a delay in disease progression [209]. The feasibility, safety, and well-tolerance of the same procedure were further confirmed by another clinical trial in a larger cohort of ALS patients [210].

Bone marrow mononuclear cells (BM-MCs) were also used for ALS treatment. A phase I clinical trial was designed in Spain to evaluate the feasibility and safety of intraspinal infusion of autologous BM-MCs [211,212]. According to neurological scales and functional respiratory indexes, the majority of side effects were minor and temporary, and no acceleration of the disease course was observed [211]. Polysomnography revealed no significant alterations in sleep quantity, quality, or breathing, indicating no malfunction of cortical diaphragmatic pathway [212]. A retrospective controlled cohort study combined intrathecal and intramuscular transplantation of autologous BM-MCs. The majority of patients reported improvements in speaking, respiration, swallowing, ambulation, and fine motor capacities, and the survival time was noticeably longer in the group that underwent cell therapy [213].

In 2009, the FDA approved the first phase 1 clinical trial for ALS, examining the safety and feasibility of intraspinal injections of NSI-566RSC, human fetal spinal cord-derived neuronal stem cells (NSCs) [214,215,216]. Additional participants were then recruited for a further phase 2 trial to test the safety of escalating doses of NSCs [217]. The procedures and doses were generally well tolerated and the majority of side effects were associated with immunosuppressant medications [217]. An Italian phase I clinical study with 18 spinal onset ALS individuals was conducted using a similar methodology [218,219]. Patients received injections of NSCs isolated and expanded from the forebrain of spontaneous miscarried fetuses. Within the first four months following transplantation, there was a considerable but brief improvement in functional status, according to ALSFRS-R scores with no side effects [219].

Bone marrow mesenchymal stem cells (BM-MSCs) are the most frequently used cell types for clinical trials in ALS despite some concerns linked to their developmental origin, the donor age, isolation method and the possibility of reintroducing the disease-related genetic defects of the patient [220]. Mazzini and colleagues performed two phase I trials to assess the feasibility and toxicity of intraspinal transplantation of autologous BM-MSCs [221,222]. Magnetic resonance imaging revealed no structural changes in either the brain or the spinal cord, also in the long term [222,223]. Despite the lack of therapeutic benefits, the results of long-term follow-up showed that the transplantation of BM-MSCs was safe and feasible [223]. Other research groups confirmed the viability and tolerability of intravenous and intrathecal injections of MSCs in patients with ALS [224,225,226,227]. Repeated intrathecal transplantation of autologous BM-MSCs obtained similar results in a phase II randomized controlled study where the treated group exhibited reduced pro-inflammatory and increased anti-inflammatory cytokines in the CSF, suggesting a possible role of MSCs in mediating the transition from pro- to anti-inflammatory states [228]. A stabilization of the disease or a reduction in the progression speed were also assessed using the ALSFRS-R score and the FVC (%). The majority of studies reported a trend toward a transitory benefit in slowing the disease progression, although these results are not definitive due to the limited size of samples and the individual variability of ALS progression [222,225,226,227,228,229,230]. Transplantation of MSCs from other sources, including adipose tissue [231] and umbilical cord Wharton’s jelly [232] was also found to be secure and well tolerated.

## 11. Conclusions

The ALS research has shed light on the intricate processes involved in muscle degeneration, emphasizing their critical role in maintaining cellular homeostasis and contributing to disease progression. The disruption of protein degradation mechanisms, such as UPS and autophagy, results in the accumulation of misfolded proteins and the formation of protein aggregates [60]. Muscle degeneration is exacerbated further by dysregulation of certain autophagy components. Additionally, oxidative stress and mitochondrial dysfunction have been linked to ALS-related muscle damage [233]. Impaired ROS detoxification and mitochondrial DNA damage worsen oxidative stress and disrupt cellular energy production. The accumulation of protein aggregates in muscle cells further contributes to oxidative stress and disrupts cellular functions [234]. The research of ALS-related muscle degeneration provides valuable insights into the underlying mechanisms of the disease and potential targets for therapeutic interventions. Understanding the dysregulation of protein degradation, autophagy, oxidative stress, and mitochondrial dysfunction is crucial for developing effective treatments and mitigating the debilitating effects of muscle degeneration in ALS patients.

NMJ has also been identified as an important component in ALS pathogenesis [90]. Axonopathy causes MN degeneration and inhibits muscle reinnervation by destroying the NMJ. Alterations in pre- and post-synaptic components of the NMJ, including TSCs, kranocytes, and signaling pathways, destabilize the NMJ and contribute to the progression of the disease [48]. In ALS, preserving NMJ function and structure represents an important therapeutic strategy. 

SCs, which are responsible for muscle regeneration, exhibit dysfunctions in ALS, resulting in impaired myotube formation and disruption of the myogenic program [108]. RNA metabolism dysregulation, notably involving RBPs such as TDP-43 and FUS, leads to the skeletal muscle degeneration seen in ALS [235]. 

Physical exercise, especially moderate-intensity activity, has also emerged as a potential therapeutic option for ALS [236]. It stimulates muscle metabolism pathways, promotes muscle regeneration, and improves muscle conditions [130]. Specific compounds, dietary programs, and hormone modulation have also been demonstrated to have potential therapeutic benefits in ALS-affected muscles [185,197]. 

In conclusion, understanding the complex interactions and underlying mechanisms involved in muscle degeneration, NMJ stability, muscle regeneration, and RNA metabolism is crucial for developing effective therapeutic interventions in ALS. Continued research is required to unravel the complexities of these processes, identify novel strategies, and improve the quality of life for ALS patients.

## Figures and Tables

**Table 1:** Main treatment strategies for muscle in ALS.

Compound	Administration	Effect	Model
Creatine	Oral	- Reduction: oxidative stress, mitochondrial dysfunction, cell death and muscle atrophy - Increase: cellular functions	Mouse SOD1-G93A/ALS patients
L-Carnitine	Injection	- Reduction: muscle death and motor neuron loss	Mouse SOD1-G93A/ALS patients
Diet	Oral	- Reduction: denervation and motor neuron loss - Increase in life	Mouse SOD1-G93A
IGF-1	Muscle expression	- Reduction: ubiquitin expression, caspase activity, protein aggregation	SOD1-G93A
Autophagy treatment	Muscle expression	- Reduction: ubiquitin expression, protein aggregation, neuronal cell death, muscle atrophy	Mouse SOD1-G93A/ALS patients
Hormone therapy	Oral	- Reduction: oxidative stress, mitochondrial dysfunction, cell death and muscle atrophy - Increase quality of life	Mouse
Palmitoylethanolamide (PEA)	Injection	- Improvements in pulmonary function, NMJ functionality and quality of life	ALS patients

## Data Availability

Not applicable.
